# How an Opportunistic Infection Can Mess with Your Brain and Take Your Breath Away: A Rare Case of Simultaneous Lung and Brain Abscess due to *Streptococcus anginosus*

**DOI:** 10.1155/2015/462459

**Published:** 2015-04-01

**Authors:** Farah Al-Saffar, Daisy Torres-Miranda, Saif Ibrahim, Adil Shujaat

**Affiliations:** ^1^Department of Medicine, University of Florida-Jacksonville, 651-3 W. 8th Street, L-18, Jacksonville, FL 32209, USA; ^2^Division of Pulmonary and Critical Care Medicine, University of Florida-Jacksonville, 651-3 W. 8th Street, L-18, Jacksonville, FL 32209, USA

## Abstract

*Streptococcus anginosus* (*S. anginosus*) is considered a friendly bug and is a one of many different bacteria that constitute the normal flora of the oral cavity. Nevertheless, it has been infrequently associated with more invasive infections, like lung abscess. It is extremely rare to have multisystemic involvement with *S. anginosus* group. We present a unique case of pulmonary and brain abscess due to *S. anginosus* in an immunocompetent patient.

## 1. Introduction

*Streptococcus anginosus* (*S. anginosus*), previously also known for one of its subgroups (*Streptococcus milleri*), a normal commensal in humans, has been commonly associated with oral infections. It has recently been reclassified under the anginosus group of* Streptococcus* [1]. More severe involvement, namely, pulmonary cavitary pneumonia with hematological spread leading to brain abscess, is very rare. There have been few cases documenting multiorgan involvement due to* S. anginosus*, even fewer to report simultaneous central nervous system and pulmonary infection [2, 3]. Interestingly, a recent report by Sunwoo and Miller Jr. recognizes how* S. anginosus* (especially in the rarity of pulmonary infections) has the bacterial potential to locally invade and consequently disseminate to different organs [4]. We present such a case, emphasizing the need to readily identify and treat* S. anginosus* given its excellent susceptibility to antibiotics.

## 2. Case Presentation

A 30-year-old male with a past medical history of asthma presented with a 3-day history of headache. He had a gradual, unrelenting headache with photophobia that was not relieved with over-the-counter pain killers. The pain was 10/10, all over the head, associated with white spots before the left eye. There was No double vision, blurring, nausea, vomiting, neck pain, fever, chills, syncope, dizziness, or trauma. The patient was admitted to a different hospital two weeks prior to presentation for pneumonia and was prescribed antibiotics for 14 days that he does not recall the name of vital signs: blood pressure was 119/55, pulse rate was 65/minute, temperature was 37.4°C (99.3°F), and oxygen saturation was normal at 96% on room air.

Physical examination was unremarkable except for fundoscopic examination that was positive for engorged retinal veins, blurred disc margins, and sluggish venous pulsation. The remainder of the neurological exam was normal. Complete blood count (CBC) and basic metabolic panel were within normal ranges.

**Figure 1:**
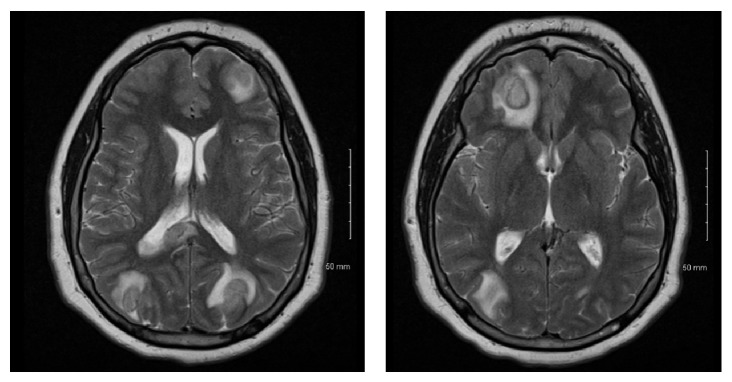
Brain MRI with contrast showing multiple ring-enhancing lesions and leptomeningeal enhancement.

**Figure 2:**
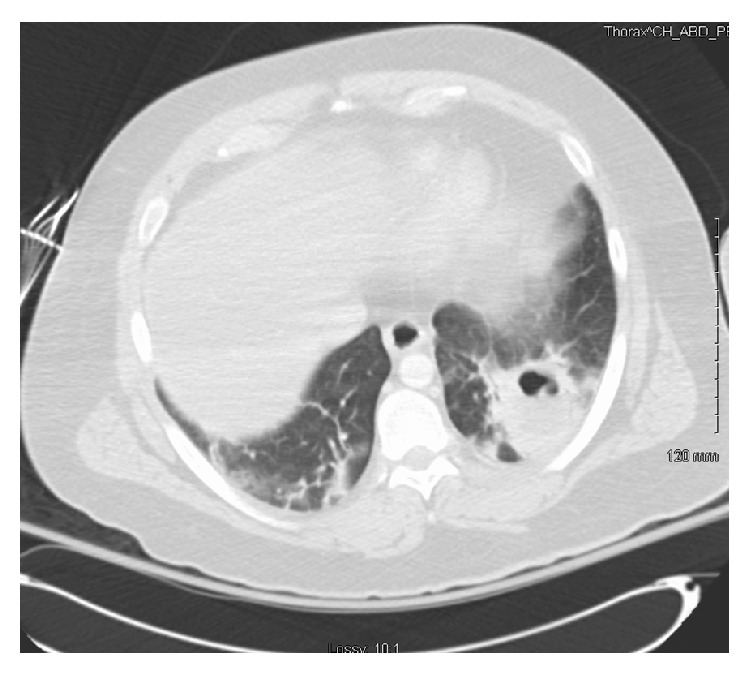
CT of the chest where left lower lobe cavitary lesion is evident.

MRI of the brain showed multifocal intraparenchymal rim-enhancing lesions and leptomeningeal enhancement, with the top differential considerations of infectious versus neoplastic etiologies (Figure 1). It also showed effacement of the fourth ventricle and basal cisterns without evidence of hydrocephalus. Chest CT showed left lower lobe cavitary lesion (Figure 2). Subsequent testing for HIV and TB was negative. His brain abscess was biopsied and cultures were positive for* S. anginosus* that was sensitive to vancomycin (which he received intrathecally) and ceftriaxone (received intravenously).

The patient initially responded to antibiotic treatment with improvement in both neurological and pulmonary functions as he was able to follow commands and could tolerate tracheostomy collar with minimal ventilator support. Unfortunately his clinical course was complicated by an extensive venous thromboembolism that led to compartment syndrome and circulatory collapse despite urgent exploration and fasciotomy and he passed away shortly afterwards.

The autopsy report confirmed multiple brain abscesses in the inferior portion of bilateral frontal lobes and bilateral parietal-occipital junction with extension into the posterior horn of the right lateral ventricle and posterior medial part of left cerebellum. It also confirmed a lung abscess in the left lower lung lobe which was the most likely the source of brain abscesses from hematogenous dissemination or retrograde flow through the vertebral veins of the causative organism (*S. anginosus*).

## 3. Discussion and Conclusion

Timely treatment for body abscesses is essential to hinder dissemination of infection and to prevent the grave complications of overwhelming sepsis and multiorgan failure. This, however, should not distract physicians from pursuing specific organism identification. Not only does it help establish the possible source of infection, but also it serves as a guideline for treatment should the patient develop relapse or reinfection later on [2, 4].

While* S. anginosus* is one of the normal flora bacteria, it can still cause extensive disseminated infections even in immunocompetent patients. The typical treatment for these infections ampicillin or vancomycin, and drainage of any concomitant abscess [2]. Proceeding respiratory tract infection has been reported in some of the previously published cases and can be a predisposing factor to subsequent hematological spread [2, 4]. Individual cultures and sensitivities are essential to individually tailor the treatment though, and residual neurological damage is common when central nervous system is involved [2]. Only few cases have been reported to date, our case being one. Immediate identification and treatment are key to the management.
